# Structural basis of ZP2-targeted female nonhormonal contraception

**DOI:** 10.1073/pnas.2426057122

**Published:** 2025-04-11

**Authors:** Elisa Dioguardi, Alena Stsiapanava, Eileen Fahrenkamp, Ling Han, Daniele de Sanctis, José Inzunza, Luca Jovine

**Affiliations:** ^a^Department of Medicine, Karolinska Institutet, Huddinge 14183, Sweden; ^b^European Synchrotron Radiation Facility—The European Synchrotron, Grenoble 38000, France; ^c^Department of Laboratory Medicine, Karolinska Institutet, Huddinge 14152, Sweden

**Keywords:** zona pellucida, ZP2, monoclonal antibody, X-ray crystallography, nonhormonal contraception

## Abstract

Monoclonal antibody IE-3 prevents mouse fertilization by binding ZP2, a major component of the oocyte-specific zona pellucida (ZP). We show that an IE-3-derived single-chain variable fragment (scFV) is sufficient for blocking fertilization in vitro and determine the structural basis of IE-3/ZP2 recognition. The high affinity of this interaction depends on induced fit of the epitope, offering insights for nonhormonal contraceptive design without off-target effects.

Due to significant technological advances, there is a renewed interest in developing contraceptive antibodies that prevent fertilization by targeting gamete interaction proteins (1). As the first interface between egg and sperm (2), the ZP has long been explored as a target for immunocontraception (3), with notable proof-of-principle applications to wildlife control (4, 5). Among the monoclonal antibodies raised against ZP proteins, of particular interest is IE-3, which targets the N-terminal ZP-N1 domain of mouse ZP2 (mZP2-N1) (6, 7). This domain was suggested to directly mediate sperm binding to the ZP (7) and implicated in the block to polyspermy by regulating the postfertilization cleavage of ZP2 (8). IE-3 binds to the ZP in vitro and in vivo (9–11). It has a full contraceptive effect in mice, with a reversible block of animal fertility (9) and no interference with normal follicular maturation, even when recombinant full-length IE-3 is expressed for many months following adeno-associated-virus-based intramuscular delivery (10). These properties establish IE-3 as a solid foundation for developing nonhormonal contraceptives. However, how the antibody exactly recognizes ZP2 at the molecular level is unknown and, more generally, there is no structural information on any antibody/female fertilization protein complex.

## Results and Discussion

**Fig. 1.**
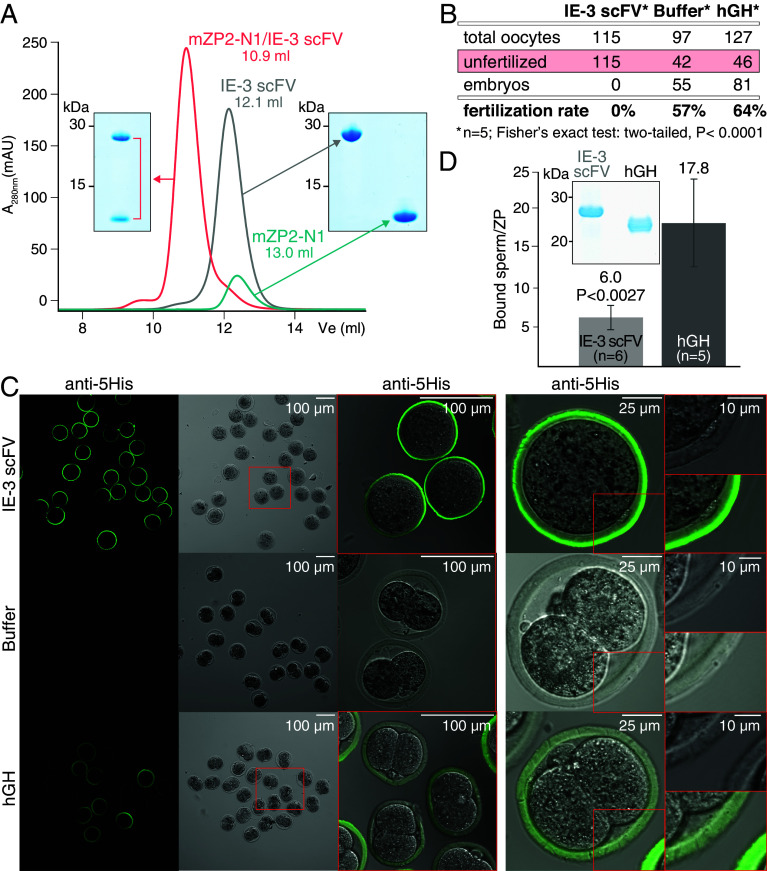
IE-3 scFV binds recombinant mZP2-N1 and blocks fertilization in vitro. (*A*) SEC analysis of purified mZP2-N1, scFV, or their complex. V_e_, elution volume. (*B*) Fertilization rates of oocytes incubated with scFV, buffer, or hGH. (*C*) Anti-5His immunofluorescent staining of representative scFV-, buffer- or hGH-treated oocytes used for the IVF experiments summarized in panel *B*. hGH-treated oocytes show a weak fluorescent signal due nonspecific protein binding to the ZP. Only oocytes incubated with buffer or hGH developed into 2-cell embryos. (*D*) Number of ZP-bound sperm after oocyte treatment with purified scFV or hGH (*Inset*).

To study how IE-3 recognizes its epitope on ZP2, we sequenced the antibody gene from its hybridoma line and engineered a His-tagged scFV that was expressed in mammalian cells and purified by immobilized metal-ion affinity chromatography (IMAC). Size-exclusion chromatography (SEC) showed that scFV forms a stable complex with mZP2-N1 in solution (Fig. 1*A*). In vitro fertilization (IVF) experiments showed that oocytes preincubated with scFV remained unfertilized, whereas oocytes exposed to buffer or human growth hormone (hGH; a control His-tagged glycoprotein of comparable molecular weight) developed into embryos (Fig. 1 *B* and *C*). Notably, scFV did not affect sperm motility but blocked fertilization by both reducing the attachment of sperm (Fig. 1*D*) and hindering ZP penetration, as suggested by the lack of spermatozoa within the ZP or in the perivitelline space (Fig. 1*C*).

**Fig. 2.**
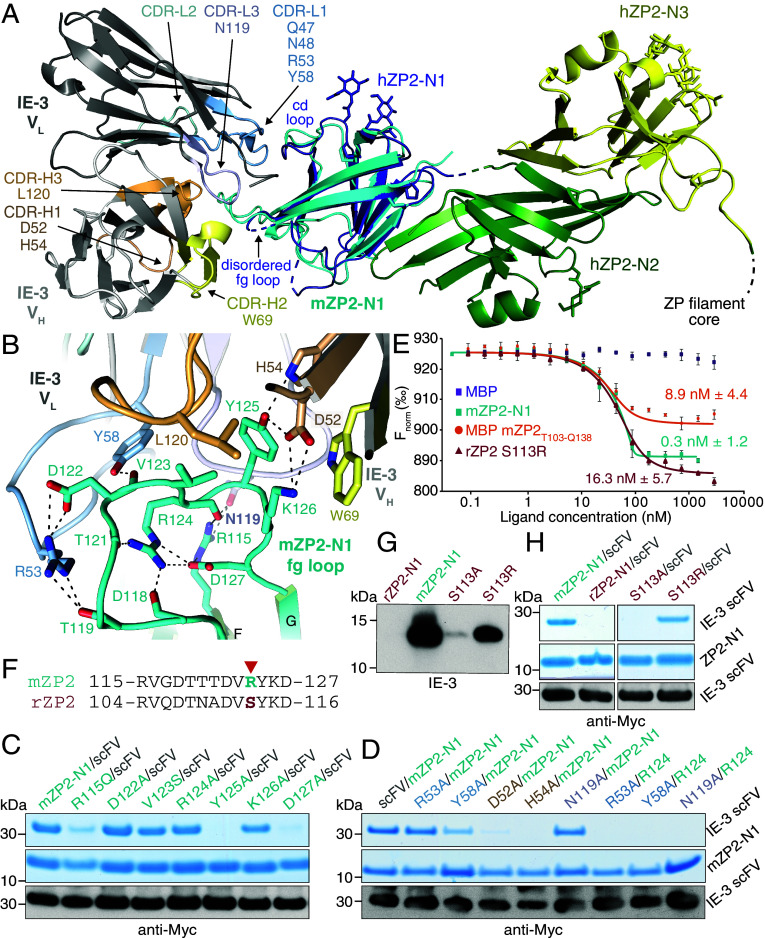
Structural analysis of mZP2-N1 recognition by IE-3. (*A*) Superposition of the mZP2-N1/V_H_V_L_ complex structure onto hZP2-N1N2N3 (PDB ID: 8RKE) with RMSD of 1.25 Å across 78 aligned residues. Selected CDRs at the interface are indicated. (*B*) Detail of mZP2-N1 fg loop/V_H_V_L_ CDR interactions. Dashed lines indicate hydrogen bonds; water molecules are omitted for clarity. (*C* and *D*) Coomassie-stained SDS-PAGE analysis of pull-down experiments using mZP2-N1 mutants (*C*) or scFV mutants and mZP2-N1/scFV double mutants (*D*). Immunoblots display the respective scFV input levels (100 µl). (*E*) Microscale thermophoresis (MST) analysis of the interaction between the fragment antigen-binding (Fab) region of IE-3 and mZP2/rZP2 constructs. MBP was used as a negative control. Kd values are reported (error bars = SD; n = 3). (*F*) Sequence alignment of rodent ZP2-N1 fg loops. A red arrow indicates mZP2 R124 and rZP2 S113. (*G*) While wild-type rZP2-N1 does not bind IE-3, the S113R mutant shows significant binding. (*H*) Pull-downs of rZP2-N1 mutants show that S113R forms a stable complex with IE-3 scFV.

To structurally investigate antibody–protein recognition, we coexpressed the unfused variable heavy and light chains of IE-3 (V_H_, V_L_) together with His-tagged mZP2-N1. This enabled efficient secretion and purification of the trimeric complex, which produced two crystal forms whose structures were determined to 1.5 Å and 2.1 Å resolution, respectively. The corresponding models are essentially identical (RMSD 0.28 Å) and their superposition onto the structure of the full N-terminal region of human ZP2 (hZP2 NTR) (8) shows that, consistent with the ability of IE-3 to recognize ZP2 in native conditions (12), the V_H_V_L_ heterodimer binds to the surface of ZP2-N1 opposite to its junction with the rest of the molecule (Fig. 2*A*). The three complementarity-determining regions of V_H_ (CDR-H1/H2/H3), together with CDRs L1 and L3 of V_L_, generate a hydrophobic pocket where the fg loop of mZP2-N1 inserts. This dominates the interaction with IE-3, which includes only a few other contacts between the cd loop of mZP2-N1 and CDR-L1 (Fig. 2*A*).

Notably, the long fg loop of ZP2 is largely disordered in the structures of both unbound mZP2-N1 (13) and hZP2 NTR (8) and becomes fixed upon interaction with the antibody (Fig. 2 *A* and *B*). Moreover, the IE-3-interacting region of the fg loop includes residues _123_VRYK_126_, which match the sequence VxYK that was previously identified as a minimal consensus epitope for IE-3 by screening a random peptide library (6). In agreement with this study, the structure of the complex and binding assays of point mutants show that hydrogen bonding of mZP2-N1 Y125 to CDR-H1 D52 and H54 is crucial for epitope recognition. On the other hand, mutation of fg loop K126, which makes a cation–π interaction with CDR-H2 W69 and also hydrogen bonds to CDR-H1 D52, does not affect complex formation (Fig. 2 *B*–*D*). Notably, the structure reveals that F-β-strand R115 and fg loop D122, two residues located outside the VxYK motif, also contribute to mZP2-N1 recognition by contacting CDR-L3 N119 and CDR-L1 R53, respectively. Most importantly, the mZP2-N1/IE-3 interaction indirectly depends on D127, which does not contact the antibody but orients R115 as well as influences the overall conformation of the fg loop by stabilizing a set of interactions centered around R124 (Fig. 2*B*). Accordingly, complex formation is significantly reduced by either individually mutating mZP2-N1 R115 or D127, or combining a mZP2-N R124A substitution with a mutation of V_L_ R53, Y58, or N119 (with each of these mutations not being sufficient to disrupt binding by itself) (Fig. 2 *C* and *D*). Consistent with an important role of the 3D shape of the fg loop, IE-3 binds mZP2-N1 with 30-fold higher affinity than the mZP2_T103-Q138_ linear epitope fused to maltose-binding protein (MBP) (Fig. 2*E*). Interestingly, IE-3 does not bind to the ZP2-N1 domain of rat ZP2 (rZP2), which contains all the aforementioned key residues except for a Ser at position 113, corresponding to mZP2-N1 R124 in the VxYK motif. However, in agreement with the above observations, mutation of S113 to Arg (but not Ala) allows rZP2-N1 to also be recognized by IE-3 scFV, albeit with reduced affinity (Fig. 2 *E*–*H*).

Our results have three main implications for the development of ZP2-targeted contraceptives. First, the finding that, despite its relatively small size, an IE-3 derived scFv is sufficient to block fertilization in vitro identifies a framework that will intrinsically avoid potential Fc-associated side effects. Second, the high-resolution structural information on mZP2-N1/V_H_V_L_, reveals that the 3D conformation of the epitope contributes significantly to its high-affinity recognition by the antibody. Consistent with the fact that IE-3 was obtained by immunization using native ZPs (12), this underlines the importance to use properly folded ZP2 material as antigen, as opposed to using linear ZP2-derived peptide sequences (6) or heat-solubilized pig ZP material (4). Third, the structure shows that IE-3 CDR-L1 makes auxiliary contacts with the cd loop of mZP2-N1. Although our experiments exclude a requirement for these interactions, rational design of CDR-L1 variants could be used to further increase the affinity for ZP2. Together, these considerations suggest that, even though the details of the mZP2/IE-3 interaction cannot be extrapolated to species with a significantly different ZP2-N1 fg loop sequence, a similar experimental approach may be used for developing nonhormonal contraception in humans [whose ZP2 NTR can be recombinantly expressed in relatively large amounts (8)] and other mammals.

## Materials and Methods

Proteins were expressed in human embryonic kidney 293 cells and purified by IMAC and SEC. IVF assays were performed using oocytes from juvenile C57BL6/J female mice and cauda epididymis sperm from mature male mice. Diffraction data were collected at beamline ID29, European Synchrotron Radiation Facility (France). Binding affinities were determined by MST. A full description of the methodology is detailed in *SI Appendix*, *Materials and Methods*.

## Supplementary Material

Appendix 01 (PDF)

## Data Availability

Sequences, structure factors and model coordinates data have been deposited in GenBank; Protein Data Bank (PDB) [MH212328, MH212329; 9H4R (14), 9H4S (15)]. All study data are included in the article and/or *SI Appendix*.
