# Supplementary material for: Structural basis of ZP2-targeted female nonhormonal contraception
Source: Proc Natl Acad Sci U S A. 2025 Apr 11;122(15):e2426057122. doi: 10.1073/pnas.2426057122 (PMC12012474; doi:10.1073/pnas.2426057122)
Supplement: Supplementary file 1 — Appendix 01 (PDF) [file pnas.2426057122.sapp.pdf]

Supplementary Information for

Structural basis of ZP2-targeted female non-hormonal contraception

Elisa Dioguardi[a,d1], Alena Stsiapanava[a1], Eileen Fahrenkamp[a,e], Ling Han[a], Daniele de Sanctis[b], José Inzunza[c], Luca Jovine[a*]

[a]Department of Medicine, Karolinska Institutet, 14183 Huddinge, Sweden
[b]ESRF – The European Synchrotron, 38000 Grenoble, France
[c]Department of Laboratory Medicine, Karolinska Institutet, 14152 Huddinge, Sweden
[d]Current affiliation: Chiesi Pharma AB, Scheeles väg 2, 17165 Solna, Sweden
[e]Current affiliation: Eurofins BioPharma Product Testing Munich GmbH, Behringstraße 6/8, 82152 Planegg, Germany
[1]E.D. and A.S. contributed equally to this work.
[*]To whom correspondence may be addressed: Luca Jovine. Address: Department of Medicine, Karolinska Institutet, 14183 Huddinge, Sweden. Phone number: +46701497014. Email: luca.jovine@ki.se.

ORCID: E.D.: 0000-0002-8734-8178; A.S.: 0000-0001-6560-011X; E.F.: 0000-0003-3482-1256; L.H.: 0000-0001-9310-4789; D.d.S.: 0000-0003-0391-8290; J.I.: 0000-0003-0876-6767; L.J.: 0000-0002-2679-6946

This PDF file includes:

Supplementary Materials and Methods

Supplementary Materials and Methods

DNA constructs

The details of the mZP2-N1 expression construct used in this study have been described previously (1). For producing additional ZP2 proteins used for binding assays, a construct encoding C-terminally 6His-tagged rZP2-N1 ($rZP2_{M1-Q127}$ with N-glycosylation site mutation N72S) was synthesized (Life Technologies/Thermo Fisher Scientific) and cloned into mammalian expression vector pHLsec3 (2); additionally, a cDNA fragment encoding mZP2 T103-Q138, essentially corresponding to the protein's B cell epitope (3), was subcloned in frame with the MBP ORF of pLJMBP6 (4).

The cDNAs for the IE-3 heavy and light chains (HC/LC) were amplified by RT-PCR from hybridoma cell line CRL-2463 (5) (ATCC) and sequenced (GenScript); the corresponding sequences have been deposited with GenBank under accession numbers MH212328 and MH212329, respectively. Note that the V_L sequence used in this study differs from that included in a previously described synthetic construct (6) at 5 positions (T71, H75, W76, Q81 and S82, corresponding to GenBank entry ALJ49777 K579, S583, L584, E589 and T590, respectively). Although none of these residues is in contact with ZP2, W76 stabilizes the V_H/V_L interface by interacting with V_H Y122, at the periphery of the hydrophobic pocket that binds the mZP2-N1 fg loop.

For IE-3 Fab expression, cDNAs encoding $HC_{M1-R239}$ followed by a 6His-tag and $LC_{M1-C240}$ were cloned into pHLsec3; for IE-3 V_HV_L expression, $HC_{Q20-S139}$ and $LC_{D21-R134}$ were cloned in frame with the Crypα signal peptide of pHLsec3. To generate the construct encoding IE-3 scFV, V_L and V_H sequences were connected by a 16-residue $(GGGS)_4$

linker and followed by 6His or Myc tags. For expression of the C-terminally His-tagged hGH control protein, the corresponding gene insert was amplified by PCR from pSGHV0 (7) and cloned into pHLsec3.

All mutations were introduced by overlapping PCR. Oligonucleotides were ordered from Sigma-Aldrich and all constructs were verified by DNA sequencing (Eurofins Genomics).

Protein expression

Proteins were transiently expressed in human embryonic kidney (HEK) 293 cells using 25 kDa branched polyethylenimine (2). For co-expression experiments of mZP2-N1 and IE-3 V_HV_L in HEK293T cells, 3 ng/µl mZP2-N1 DNA were mixed with 3.5 ng/µl DNA V_H and 3.5 ng/µl DNA V_L; all other co-transfections were performed using a 1:1 DNA ratio.

MBP-mZP2$_{T103\text{-}Q138}$ and control MBP were expressed in *E. coli* BL21 pLysS (DE3) (Promega) at 21ºC. Protein expression was induced for 16–18 h with 0.1 mM IPTG at an optical density (OD_{550}) of 0.5–1. Cells from 1 L culture, suspended in 10 mL 50 mM Tris–HCl pH 7.5, 50 mM NaCl, 1 mM $MgCl_2$, 0.2 mg mL^{-1} lysozyme, 25 U mL^{-1} Benzonase (Sigma-Aldrich) and cOmplete mini EDTA-free protease inhibitors (Roche), were disrupted using three freeze-thaw cycles. Bacterial debris was removed by centrifugation at 18,000 x g for 30 min.

Protein purification

72 h after transfection, the conditioned media from mammalian cells was harvested, 0.22 µm-filtered (Sarstedt) and adjusted to 20 mM Na-HEPES pH 7.8, 500 mM NaCl, 5-10 mM

imidazole (IMAC buffer). 10 mL pre-equilibrated nickel agarose slurry (Ni-NTA; QIAGEN) was added per liter of medium and incubated for 1 h at room temperature (RT). After washing the beads with 100 column volumes IMAC buffer, proteins were batch-eluted with 5 column volumes elution buffer (20 mM Na-HEPES pH 7.8, 150 mM NaCl, 500 mM imidazole). Proteins were concentrated with appropriate centrifugal filtration devices (Amicon) and further purified by SEC at 4ºC using an $ÄKTA_{FPLC}$ chromatography system (GE Healthcare). mZP2-N1/IE-3 V_HV_L complex was injected into a Superdex 75 26/60 column (GE Healthcare) pre-equilibrated with 10 mM Tris-HCl pH 7.2, 50 mM NaCl. Peak fractions were pooled and the protein complex was concentrated to 27 mg mL^{-1} for crystallization trials.

Batch IMAC of $MBP\text{-}mZP2_{T103\text{-}Q138}$ and MBP were carried out for 1 h at RT in 50 mM Tris-HCl pH 7.5, 1 M NaCl, 20 mM imidazole; proteins were eluted with 50 mM Tris-HCl pH 7.5, 150 mM NaCl, 500 mM Imidazole and further polished by SEC using a Superdex 200 26/600 column (GE Healthcare) pre-equilibrated with 10 mM Na-HEPES pH 7.5, 150 mM NaCl.

Protein analysis

For immunoblotting, proteins separated by SDS-PAGE were transferred to a nitrocellulose membrane (GE Healthcare Life Sciences) and probed with the following primary antibodies: anti-mZP2 IE-3 monoclonal (1:1,000); anti-5His monoclonal (1:1,000; QIAGEN); anti-Myc monoclonal (1:1,000; Sigma-Aldrich clone 9E10). Secondary antibodies were goat anti-rat (1:10,000; Thermo Fisher Scientific); horseradish peroxidase-conjugated goat anti-mouse (1:10,000) (Life Technologies/Thermo Fisher

Scientific). Chemiluminescence detection was performed with Western Lightning ECL Plus (Perkin Elmer).

Pull-down analysis of protein-protein interaction

2 mL conditioned medium containing 6His-tagged mZP2-N1 were harvested three days post-transfection and centrifuged for 5 min at 500 x g. 20 mM Na-HEPES pH 7.8, 150 mM NaCl and 5 mM imidazole were added and samples were incubated with 50 µL Ni-NTA beads for 1 h at RT. Beads were collected by centrifugation at 100 x g and washed 3 times with 1 mL 20 mM Na-HEPES pH 7.8, 150 mM NaCl, 10 mM imidazole. Bound material was eluted with 100 µL 20 mM Na-HEPES pH 7.8, 150 mM NaCl, 500 mM imidazole and 20 µL were analyzed by immunoblot or Coomassie blue staining.

Binding affinity determination by microscale thermophoresis

MST analysis was performed using a NanoTemper Monolith NT.115 instrument (NanoTemper Technologies). Because of severe bleaching of IE-3 scFV, recombinantly expressed IE-3 Fab was used and labeled with the Blue-NHS labeling kit (NanoTemper Technologies GmbH), according to the manufacturer's instructions and using a labeling buffer containing 10 mM Na-HEPES pH 7.5, 150 mM NaCl, 0.01% (v/v) Tween 20. Varying concentration of mZP2-N1, rZP2-N1 S113R and MBP-mZP2$_{T103\text{-}Q138}$ in labeling buffer were titrated against labeled IE-3 Fab (8 nM) in 20 mM Na-HEPES pH 7.8, 200 mM NaCl, 0.01% (v/v) Tween 20. Unfused MBP was used as negative control. Samples were loaded into Premium Coated Capillaries (NanoTemper Technologies) and measurements were performed using 20% MST power and 85% LED power. Laser-on

and -off times were 30 s and 5 s, respectively. Each of the graphs superimposed in Fig. 2E displays merged data from three independent experiments. Datasets were processed with the MO.Affinity Analysis software (NanoTemper Technologies) using the signal from the thermophoresis T jump.

Protein crystallization

Crystallization experiments were performed by hanging drop vapor diffusion at 20ºC using a mosquito crystallization robot (TTP Labtech) using a Nunc 96 multiwell plate (Thermo Scientific) with a ratio protein:reservoir 1:1 (drop size 200 nL). The mZP2-N1/IE-3 V_HV_L complex crystallized in 24% (v/v) PEG 3350, 0.2 M sodium tartrate, 0.1 M Na-HEPES pH 6.5 (crystal form I; $P2_12_12_1$) as well as 2.0 M lithium sulfate, 0.2 M ammonium sulfate, 0.1 M tri-sodium citrate pH 5.6 (crystal form II; $P4_3$).

X-ray diffraction data collection

Crystals were cryoprotected using mother liquor solutions supplemented with 15% (v/v) PEG 200 (crystal form I) or 10% (v/v) PEG 200 (crystal form II).

All datasets were collected from single crystals at 100 K. The initial orthorhombic dataset of mZP2-N1/IE-3 V_HV_L was collected in house using a Compact HomeLab system (Rigaku) equipped with a PILATUS 200K detector (DECTRIS), while final datasets for both crystal forms were collected at a wavelength of 0.9763 Å at European Synchrotron Radiation Facility (ESRF) beamline ID29 (8), using a PILATUS 6M-F detector (DECTRIS). For crystal form I, data collection statistics generated using phenix.table_one (9) were: completeness 93.4 (83.8) %, multiplicity 3.3 (3.4), mean I/sigma(I) 20.2 (1.1),

R_{merge} 0.025 (1.166), R_{meas} 0.030 (1.375), R_{pim} 0.016 (0.720), $CC_{1/2}$ 1.00 (0.54), CC* 1.00 (0.84) (with the values in parentheses being for the highest resolution shell, 1.58-1.53 Å). For crystal form II, statistics were: completeness 97.3 (92.5) %, multiplicity 6.8 (7.1), mean I/sigma(I) 26.1 (1.5), R_{merge} 0.052 (1.369), R_{meas} 0.056 (1.478), R_{pim} 0.021 (0.553), $CC_{1/2}$ 1 (0.53), CC* 1.00 (0.83) (with the values in parentheses being for the highest resolution shell, 2.09 - 2.02 Å).

Data processing and structure determination

X-ray diffraction datasets were processed using XDS (10). A 2.30 Å resolution dataset collected in house was used to determine the orthorhombic structure of the mZP2-N1/IE-3 V_HV_L complex by molecular replacement (MR) with Phaser (11), using as independent search models the structure of unbound mZP2-N1 (PDB ID 5II6, chain A residues P43-Q138) (1) and those of the V_H domain of anti-porphyrin antibody 13G10 (PDB ID 4AMK, chain H residues Q3-S118) (12) and the V_L domain of chimeric antibody X836 (PDB ID 3MBX, chain L residues D1-R114) (13), both of which were pre-processed with Sculptor (14). An initial model, consisting of two copies of the complex related by non-crystallographic symmetry (NCS) (corresponding to mZP2-N1, V_H, V_L chains A, H, L and B, X, Y, respectively), was autotraced with PHENIX AutoBuild (15). After manual rebuilding in Coot (16), the model was refined to R/R_{free} 0.206/0.233 against the 1.53 Å resolution synchrotron dataset using phenix.refine (17) (with torsion-based NCS restraints, as well as twinning operator k, h, -l and twinning fraction 0.3). Protein geometry and all-atom contacts were validated with MolProbity (18): Ramachandran

favored/allowed/outliers: 98.2/1.8/0.0 %; Rama distribution Z-score: 0.53 ± 0.31; favored/poor rotamers: 96.5/0.0 %; MolProbity score: 1.12; clashscore 3.28.

The tetragonal structure of mZP2-N1/IE-3 V_HV_L, whose asymmetric unit also includes two complexes, was solved by MR using a partially refined model of the $P2_12_12_1$ structure. The solution was rebuilt, refined to R/R_{free} 0.189/0.203 at 2.02 Å resolution and validated as above (Ramachandran favored/allowed/outliers: 98.0/2.0/0.0 %; Rama distribution Z-score: -0.08 ± 0.31; favored/poor rotamers: 98.1/0.2 %; MolProbity score: 1.00; clashscore 2.24). The average B-factor of V_L (chain L) in the orthorhombic structure of the complex is significantly higher than that of the other chains (50 Å^2 vs 34 Å^2); similarly, the average B-factor of V_H (chain H) in the tetragonal structure of the complex is higher than that of the other chains (79 Å^2 vs 52 Å^2). However, in both cases, the B-factors of the ZP2/IE-3 interface residues in the AHL and BXY complexes are comparable (30 Å^2 vs 28 Å^2 (crystal form I); 45 Å^2 vs 42 Å^2 (crystal form II)).

Structural alignments were performed using Chimera (19), Coot and PyMOL (Schrödinger, LLC). Protein-protein interfaces and oligomeric states were analyzed using PIC (20) and PDBsum (21). All figures were created with PyMOL.

Mouse gamete collection

3- to 5-week old C57BL6/J female mice were used as oocyte donors. Mice were kept under controlled light and temperature conditions with free access to food and water at the Preclinical Laboratory Portal South Core Facility of Karolinska University Hospital (Huddinge, Sweden). Ovarian stimulation was induced by intraperitoneal administration of 5 IU Pregnant Mare Serum Gonadotrophin (PMSG; Folligon, Intervet) and, 48 h later,

5 IU human Chorionic Gonadotropin (hCG; Chorulon, Intervet). 12 h after the hCG injection females were sacrificed and ampullas from individual animals were retrieved and equilibrated in HTF medium (Embryo Max, Millipore). Caudae epididymis were dissected from mature male mice and placed into 1 mL drops of HTF medium supplemented with 0.4% (w/v) BSA and covered with oil (OVOIL, Vitrolife). Sperm was capacitated by incubation for 1 h at 37ºC, 5% CO_2. All animal experiments were performed in accordance with the approval of the local ethical committee (Stockholm South Animal Ethics Committee, Sweden; application number S-26-15).

In vitro fertilization assays

IE-3 scFV and control hGH, purified in 10 mM Na-HEPES pH 7.5 and 150 mM NaCl, were concentrated to 1.2 mg mL^{-1} and 1.3 mg mL^{-1}, respectively. After 15 min equilibration in HTF medium at RT, 5 µL protein was added to IVF drops containing an average of 16 oocytes surrounded by cumulus cells and covered with oil. Protein and oocytes were incubated for 1 h at 37ºC, 5% CO_2. Because the fertilization block induced by IE-3 is concentration-dependent (22), a 10-fold excess of IE-3 scFV (corresponding to 2.2 µM final protein concentration in the IVF drop) was used to ensure full saturation of its binding sites in the ZP. Control hGH was used at the same molar concentration of IE-3 scFV. After capacitation, ~ 5 x 10^6 mL^{-1} highly motile sperm was added to the IVF drops containing oocytes and proteins; 4 h later, oocytes were washed, incubated overnight, and observed to assess 2-cell cleavage occurrence. Fertilization rates were calculated based on the number of healthy cells.

To count the number of sperm attached to the ZP, 5 groups of 5-10 oocytes (hGH) or 6 groups of 5-15 oocytes (IE-3 scFV), corresponding to a total of 36 or 54 oocytes, respectively, were washed in PBS 1 h after having added capacitated sperm and fixed in 2% (v/v) paraformaldehyde (PFA; Sigma-Aldrich). Oocytes were then washed three times and finally fixed using 2-cell embryos as washing controls. Sperm was counted by performing a through-focus series on each oocyte.

Confocal microscopy

2 cell-embryos or unfertilized oocytes were fixed with 2% (v/v) PFA, washed with 100 µL PBS and blocked with a solution containing 1x PBS, 3% (w/v) BSA for 1 h at RT. After three washing steps with PBS, cells were incubated overnight at 4˚C with anti-5His monoclonal (1:1000). The day after, after three washing steps in 1x PBS, cells were transferred into a drop containing PBS supplemented with 3% BSA and goat anti-mouse IgG Alexa 488 secondary antibody (1:1000; Thermo Fisher Scientific) and incubated for 1 h at RT. Samples were washed, transferred to a glass coverslip with PBS and imaging was performed on a Nikon A1R confocal microscope at RT, at the Karolinska Institutet Live Cell Imaging Core Facility (Huddinge, Sweden).

References

1. I. Raj, *et al.*, Structural Basis of Egg Coat-Sperm Recognition at Fertilization. *Cell* **169**, 1315–1326.e17 (2017).
2. M. Bokhove, *et al.*, Easy mammalian expression and crystallography of maltose-binding protein-fused human proteins. *J. Struct. Biol.* **194**, 1–7 (2016).
3. W. Sun, Y. H. Lou, J. Dean, K. S. Tung, A contraceptive peptide vaccine targeting sulfated glycoprotein ZP2 of the mouse zona pellucida. *Biol. Reprod.* **60**, 900–907 (1999).
4. M. Monné, L. Han, T. Schwend, S. Burendahl, L. Jovine, Crystal structure of the ZP-N domain of ZP3 reveals the core fold of animal egg coats. *Nature* **456**, 653–657 (2008).
5. I. J. East, J. Dean, Monoclonal antibodies as probes of the distribution of ZP-2, the major sulfated glycoprotein of the murine zona pellucida. *J. Cell Biol.* **98**, 795–800 (1984).
6. J. Li, *et al.*, Vectored antibody gene delivery mediates long-term contraception. *Curr. Biol.* **25**, R820–R822 (2015).
7. D. J. Leahy, C. E. Dann 3rd, P. Longo, B. Perman, K. X. Ramyar, A mammalian expression vector for expression and purification of secreted proteins for structural studies. *Protein Expr. Purif.* **20**, 500–506 (2000).
8. D. de Sanctis, *et al.*, ID29: a high-intensity highly automated ESRF beamline for macromolecular crystallography experiments exploiting anomalous scattering. *J. Synchrotron Radiat.* **19**, 455–461 (2012).

9. P. D. Adams, *et al.*, PHENIX: a comprehensive Python-based system for macromolecular structure solution. *Acta Crystallogr. D Biol. Crystallogr.* **66**, 213–221 (2010).

10. W. Kabsch, XDS. *Acta Crystallogr. D Biol. Crystallogr.* **66**, 125–132 (2010).

11. A. J. McCoy, *et al.*, Phaser crystallographic software. *J. Appl. Crystallogr.* **40**, 658–674 (2007).

12. V. Muñoz Robles, *et al.*, Crystal structure of two anti-porphyrin antibodies with peroxidase activity. *PLoS One* **7**, e51128 (2012).

13. A. Teplyakov, *et al.*, On the domain pairing in chimeric antibodies. *Mol. Immunol.* **47**, 2422–2426 (2010).

14. G. Bunkóczi, R. J. Read, Improvement of molecular-replacement models with Sculptor. *Acta Crystallogr. D Biol. Crystallogr.* **67**, 303–312 (2011).

15. T. C. Terwilliger, *et al.*, Iterative model building, structure refinement and density modification with the PHENIX AutoBuild wizard. *Acta Crystallogr. D Biol. Crystallogr.* **64**, 61–69 (2008).

16. P. Emsley, B. Lohkamp, W. G. Scott, K. Cowtan, Features and development of Coot. *Acta Crystallogr. D Biol. Crystallogr.* **66**, 486–501 (2010).

17. P. V. Afonine, *et al.*, Towards automated crystallographic structure refinement with phenix.refine. *Acta Crystallogr. D Biol. Crystallogr.* **68**, 352–367 (2012).

18. V. B. Chen, *et al.*, MolProbity: all-atom structure validation for macromolecular crystallography. *Acta Crystallogr. D Biol. Crystallogr.* **66**, 12–21 (2010).

19. E. F. Pettersen, T. D. Goddard, C. C. Huang, UCSF Chimera—A visualization system for exploratory research and analysis. *J. Comput. Chem.* **25**, 1605–1612 (2004).

20. K. G. Tina, R. Bhadra, N. Srinivasan, PIC: Protein Interactions Calculator. *Nucleic Acids Res.* **35**, W473–W476 (2007).

21. R. A. Laskowski, *et al.*, PDBsum: a Web-based database of summaries and analyses of all PDB structures. *Trends Biochem. Sci.* **22**, 488–490 (1997).

22. I. J. East, D. R. Mattison, J. Dean, Monoclonal antibodies to the major protein of the murine zona pellucida: effects on fertilization and early development. *Dev. Biol.* **104**, 49–56 (1984).
